# An Application of Evidence‐Based Approaches to Engage Young People in the Design of a Global Mental Health Databank

**DOI:** 10.1111/hex.14172

**Published:** 2024-09-07

**Authors:** Augustina Mensa‐Kwao, Lakshmi Neelakantan, Jennifer Velloza, Emily Bampton, Swetha Ranganathan, Refiloe Sibisi, Joshua Bowes, Lilliana Buonasorte, Damian Omari Juma, Manasa Veluvali, Megan Doerr, Tamsin Jane Ford, Christine Suver, Carly Marten, The MindKind Consortium, Faith Oluwasemilore Adeyemi, Patricia A. Areán, Emily Bampton, Elin A. Björling, Ljubomir Bradic, Anne‐Marie Burn, Emma Grace Carey, Sonia Carlson, Tessa Concepcion, Meera Damji, Megan Doerr, Julia C. Dunbar, Mina Fazel, Blossom Fernandes, Gillian Finchilescu, Tamsin Ford, Melvyn Freeman, Isabell R. Griffith Fillipo, Jay Hodgson, Jasmine Kalha, Minal Karani, Michael R. Kellen, Christopher G. Kemp, Simthembile Lindani, Lara M. Mangravite, Carly Marten, Hedwick Masomera, Felicia Mata‐Greve, Augustina Mensa‐Kwao, Emily Moore, Erin Mounts, Lakshmi Neelakantan, Larsson Omberg, Lisa Pasquale, Soumitra Pathare, Swetha Ranganathan, Nichole Sams, Jo Scanlan, Himani Shah, Sotirios Short, Refiloe Sibisi, Solveig K. Sieberts, Stockard Simon, Sushmita Sumant, Christine Suver, Yanga Thungana, Meghasyam Tummalacherla, Chandre Van Vught, Zukiswa Zingela, Pamela Y. Collins, Jennifer Velloza

**Affiliations:** ^1^ Department of Mental Health Johns Hopkins Bloomberg School of Public Health Baltimore Maryland USA; ^2^ Population Mental Health Unit, Centre for Mental Health and Community Wellbeing, School of Population and Global Health University of Melbourne Melbourne Australia; ^3^ Department of Epidemiology & Biostatistics University of California San Francisco San Francisco California USA; ^4^ Department of Psychiatry University of Oxford Oxford UK; ^5^ Centre for Mental Health Law & Policy Indian Law Society (ILS) Pune Maharashtra India; ^6^ Activate Change Drivers ZA Johannesburg South Africa; ^7^ King's College London London UK; ^8^ Department of Anthropology and Archaeology University of Bristol Bristol UK; ^9^ Healthy Brains Global Initiative Mombasa Kenya; ^10^ Sage Bionetworks Seattle Washington USA; ^11^ Department of Psychiatry University of Cambridge Cambridge UK; ^12^ Sage Bionetworks Seattle Washington USA; ^13^ Department of Psychiatry University of Oxford Oxford Oxfordshire UK; ^14^ Human Centered Design and Engineering University of Washington Seattle Washington USA; ^15^ Department of Psychiatry University of Cambridge Cambridge Cambridgeshire UK; ^16^ Centre for Mental Health Law & Policy, Indian Law Society Pune Maharashtra India; ^17^ Department of Psychiatry Walter Sisulu University Eastern Cape South Africa; ^18^ Activate Change Drivers ZA Johannesburg Gauteng South Africa; ^19^ Department of Epidemiology & Biostatistics University of California San Francisco San Francisco California USA; ^20^ Department of Psychiatry and Behavioral Sciences University of Washington Seattle Washington USA; ^21^ University of Stellenbosch Stellenbosch Western Cape South Africa; ^22^ Nelson Mandela University Gqeberha Eastern Cape South Africa; ^23^ Department of Global Health University of Washington Seattle Washington USA; ^24^ Higher Health, Centurion Gauteng South Africa; ^25^ Cambridgeshire and Peterborough Foundation NHS Trust Fulbourn Cambridgeshire UK; ^26^ University of Johannesburg Johannesburg Gauteng South Africa; ^27^ Department of Psychology University of Bath Bath UK; ^28^ Department of Psychology University of the Witwatersrand Johannesburg Gauteng South Africa; ^29^ CREATIV Lab University of Washington Seattle Washington USA; ^30^ Department of International Health Johns Hopkins Bloomberg School of Public Health Baltimore Maryland USA; ^31^ Department of Mental Health Johns Hopkins Bloomberg School of Public Health Baltimore Maryland USA

**Keywords:** adolescent, global mental health, health technology, mental health, youth engagement

## Abstract

**Introduction:**

Engaging youth in mental health research and intervention design has the potential to improve their relevance and effectiveness. Frameworks like Roger Hart's ladder of participation, Shier's pathways to participation and Lundy's voice and influence model aim to balance power between youth and adults. Hart's Ladder, specifically, is underutilized in global mental health research, presenting new opportunities to examine power dynamics across various contexts. Drawing on Hart's ladder, our study examined youth engagement in mental health research across high‐ and middle‐income countries using Internet‐based technologies, evaluating youth involvement in decision‐making and presenting research stages that illustrate these engagements.

**Methods:**

We conducted a directed content analysis of youth engagement in the study using primary data from project documents, weekly AirTable updates and discussions and interviews with youth and the research consortium. Using Hart's Ladder as a framework, we describe youth engagement along rungs throughout different research stages: cross‐cutting research process, onboarding, formative research and quantitative and qualitative study designs.

**Results:**

Youth engagement in the MindKind study fluctuated between Rung 4 (‘Assign, but informed’) and Rung 7 (‘Youth initiated and directed’) on Hart's Ladder. Engagement was minimal in the early project stages as project structures and goals were defined, with some youth feeling that their experiences were underutilized and many decisions being adult‐led. Communication challenges and structural constraints, like tight timelines and limited budget, hindered youth engagement in highest ladder rungs. Despite these obstacles, youth engagement increased, particularly in developing recruitment strategies and in shaping data governance models and the qualitative study design. Youth helped refine research tools and protocols, resulting in moderate to substantial engagement in the later research stages.

**Conclusion:**

Our findings emphasize the value of youth–adult partnerships, which offer promise in amplifying voices and nurturing skills, leadership and inclusiveness of young people. Youth engagement in project decision‐making progressed from lower to higher rungs on Hart's Ladder over time; however, this was not linear. Effective youth engagement requires dynamic strategies, transparent communication and mutual respect, shaping outcomes that authentically reflect diverse perspectives and mental health experiences.

**Patient or Public Contribution:**

There was substantial patient and public involvement in this study. This paper reports findings on youth engagement conducted with 35 young people from India, South Africa and the United Kingdom, all of whom had lived experience of mental health challenges. Youth engagement in the MindKind study was coordinated and led by three professional youth advisors (PYAs) in these contexts, who were also young people with lived experience of mental health challenges. Each of the three study sites embedded a full‐time, community‐based PYA within their study team to inform all aspects of the research project, including the development of informational materials and the facilitation of Young People's Advisory Group (YPAG) sessions referenced in this paper. Each PYA also consulted with a site‐specific YPAG that met bi‐monthly throughout the project, shaping the formation of study materials and serving as a test group in both the quantitative and qualitative studies. Youth participants in this study also contributed extensively, engaging in data collection and manuscript writing. The following youth advisory panels members (J.B., L.B., D.O.J., M.V.) and all PYAs (E.B., S.R., R.S.) in the MindKind study contributed to the writing of this manuscript and are acknowledged as co‐authors.

## 1 Introduction

Globally, an estimated 5% and 9.3% of youth aged 15–24 years experience major depressive disorder and anxiety disorders, respectively, [1] which are associated with negative health, educational, social and economic outcomes in adulthood [2]. Participatory, youth‐oriented research can enhance the feasibility, ecological validity, value and impact of findings [3, 4], thereby improving youth mental health. Youth engagement also enhances intervention relevance and patient outcomes in clinical settings by personalizing interventions [5, 6]. Young people globally express a desire to participate in mental health research, practice and policy [7]. Participatory research includes co‐design, co‐production, and co‐creation [8], focusing on research *with*, rather than research *on*, youth [9]. While acknowledging the distinctions between these approaches, we use the term ‘youth engagement’ in this paper to highlight the active engagement of young people in this study, that is, designing a global mental health databank, detailing their interactions with researchers throughout the process [10, 11, 12, 13].

Youth engagement involves opportunities such as youth–adult partnerships [10], youth‐led participatory action research [11], young people's advisory groups (YPAGs) [12], youth advisory boards and councils [13] and digital mental health research [14]. A systematic review of youth engagement in mental health research highlighted partnerships with youth (i.e., youth work collaboratively with researchers as equals) as the most common form of engagement, followed by consultations (i.e., youth provide feedback on research); only one youth‐led study (i.e., every stage of research is driven by youth) was identified [15]. Youth involvement in digital mental health interventions often took a consultative role in design, prototyping and testing [14]. Engagement methods included individual/group meetings, workshops, nominal group technique, expert consensus methods, that is, Delphi exercises, and advisory groups.

Several frameworks exist to conceptualize youth engagement in research, practice and policy. Frameworks identifying the allocation and balance of power between youth and adults have been widely applied, for example, Roger Hart's ladder of participation [16], Shier's pathways to participation [17] and Lundy's voice and influence model [18]. Some frameworks emphasize the *process* of youth engagement and aim to address the barriers and facilitators, for example, the McCain Centre Model of Youth Engagement and the P7 model [19]. Others conceptualize the *impacts* of youth engagement in research, for example, the EIPARS model, [20] which offers a six‐step process from engagement to sustainability [21]. A few frameworks aim to promote *equity* through alternative research methods, for example, the YPAR 2.0 Model of Research Engagement [22], which positions research as a tool to co‐produce knowledge with communities [21].

Few studies comprehensively evaluate youth engagement and decision‐making in mental health research globally. Although well documented in high‐income countries (HICs) [12], evaluations in both HICs and in low‐ and middle‐income countries (LMICs) are scarce, with many relying on anecdotal evidence. A study in Australia examined different forms of youth participation, emphasizing the importance of nurturing, protecting and respecting young people, involving them in decision‐making, improving services through their perspectives and recognizing the developmental benefits of their involvement [23]. The study also noted challenges, such as excluding vulnerable or hard‐to‐reach groups and the risk of youth participation being isolated from mainstream public affairs [23]. However, it overlooked the contextual differences present in LMIC regions. There is a notable gap in understanding how youth engagement and decision‐making evolve over time in mental health research across HICs and LMICs. This gap may hinder the development of tailored interventions that consider young individuals’ evolving needs and preferences.

Unique barriers to engagement may affect marginalized youth and those in low‐resource settings. For instance, Internet access remains highly unequal, with 53% and 81% lacking internet access in developing and least developed countries, respectively, compared to only 13% in high‐income settings [24]. The COVID‐19 pandemic accelerated Internet‐based research [25] and highlighted the demand to engage diverse youth in mental health research [26]. Although general guidance exists for engaging with diverse cultural groups, such as building trustworthy relationships, few studies describe the quality of youth engagement over time and in decision‐making processes in multi‐country research [27].

Current research lacks comprehensive evaluations of youth engagement in mental health studies, particularly in LMICs. Empirical data on the evolution of youth engagement and decision‐making over time are scarce, and there is insufficient understanding of the barriers faced by marginalized youth in low‐resource settings. Our paper addresses these gaps by systematically mapping and evaluating youth engagement using a theoretical framework. Drawing on Hart's ladder as an organizing and evaluative framework, we aimed to explore youth engagement processes in mental health research conducted across three HICs and middle‐income countries using Internet‐based technologies.

**Figure 1:**
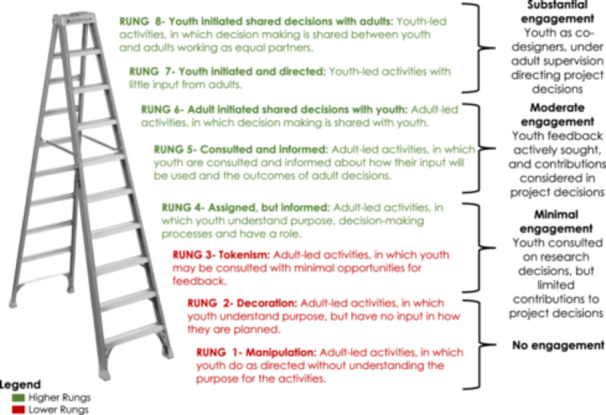
Roger Hart's ladder of participation.

We conceptualize youth engagement using Hart's ladder of participation, a widely recognized framework for youth engagement [28, 29, 30, 31]. Hart's Ladder, building on Arnstein's concept of citizen participation [32], categorizes youth engagement into eight levels, from tokenism to youth‐led decision‐making at the highest rung (Figure 1) [16]. It constructs engagement as a negotiated process that can change over time, with the need to align involvement with youths’ everyday lives [33]. Critiques of Hart's Ladder often point to its linear and hierarchical nature [19, 34], suggesting that it may not always align with the practical needs of diverse projects, which calls for adaptable engagement approaches. However, the benefits of Hart's ladder include identifying current engagement levels, providing a pathway to evaluate engagement and promoting transparency. It also serves as an educational tool for stakeholders, illustrating the shifting roles, power structures and youth contributions [35]. The use of Hart's ladder across various fields and cultural and social contexts suggests considerable utility and relevance [28, 29, 30, 31].

Despite its recognition across different fields, the application and flexibility of Hart's Ladder in global mental health research, especially in the Global South, have not been thoroughly described. Few studies map and evaluate youth engagement and decision‐making in mental health research across global contexts.

Our specific objectives were to (1) Describe and evaluate how youth were engaged in research decision‐making across the three study sites (India, South Africa and the United Kingdom) and (2) Illustrate the process of such engagement and the evolution of power allocation between youth and non‐youth stakeholders at different stages of research. Our study reports on the experience of undertaking youth engagement in the MindKind study, which tested the feasibility of designing a global mental health databank for research on young people [36, 37].

## 2 Materials and Methods

### 2.1 Study Design

The MindKind project, funded by the Wellcome Trust, aimed to create a global mental health databank using data from young people in India, South Africa and the United Kingdom [36, 37]. Participants aged 16–24 in the United Kingdom and 18–24 in South Africa and India were randomly assigned to one of four data governance models and monitored for 12 weeks. One group chose their data governance preferences before consenting, while the others received model‐specific consent and chose whether to participate. The MindKind study used a youth‐adult participatory research approach, grounded in young people's right to be involved in decisions that impact their human rights, as outlined in Article 12 of the United Nations Convention on the Rights of the Child. Additional details on the study design and objectives have been published elsewhere [36, 37].

**Figure 2:**
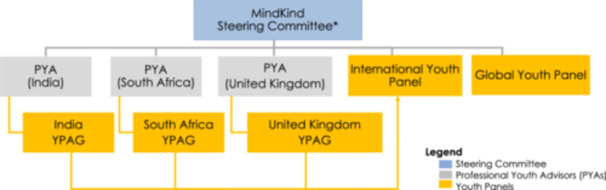
MindKind project governance and structure. *Steering committee members were representatives from all institutions involved in the project.

The project included three professional youth advisors (PYAs) and international youth panels throughout the 18‐month study period. Governance was overseen by the MindKind Steering Committee (~15 members), with PYAs having full voting rights [38]. The funder was not a member of the Steering Committee but was actively involved in the study and with youth engagement, that is, a lived experience advisor employed by the funder mentored the PYAs regularly [38]. Figure 2 illustrates the MindKind project governance. Project activities were conducted remotely due to COVID‐related constraints.

### 2.2 PYAs

We hired one PYA per country as full‐time team members (United Kingdom, India and South Africa). Further details on the PYAs involvement in MindKind study can be found elsewhere [38]. PYAs led advisory groups meetings, documented findings, facilitated capacity building and represented youth in Steering Committee meetings.

### 2.3 In‐Country YPAGs

Youth engagement in the MindKind study primarily involved 12–16 YPAG members in each country site. They were selected for diversity in various aspects (e.g., regional origin, life experiences, gender and disability status) and attended bi‐monthly meetings, providing feedback on project activities. YPAG members received honoraria (in all sites) and mobile data (in South Africa). They occasionally participated in other study activities, including manuscript writing, presentations and membership in an International Youth Panel. YPAGs contributed to data governance, data collection design and methods, and co‐authored project manuscripts.

### 2.4 Additional Youth Panels

The MindKind study established two additional panels: the Global Youth Panel (GYP) and the International Youth Panel (IYP). The GYP comprised 15 members each from three study sites and additional members from the USA, Canada, Kenya, Nigeria and South Africa [39]. The GYP primarily engaged in the formative research phase, offering high‐level feedback on project decisions to guide the future testing and expansion of the MindKind study beyond the initial sites [39]. The IYP consisted of three to five representatives from each country YPAG, with varying panel sizes. They met monthly with the University of Washington team to provide feedback on project priorities and receive capacity building, and received honoraria and data (in South Africa) for their participation [39].

#### 2.4.1 Data Collection

**Figure 3:**
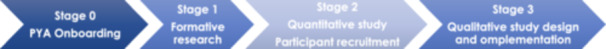
MindKind research stages.

We collected data from three study sites—India, South Africa and the United Kingdom—on PYAs and youth panel engagement throughout the 20‐month study using AirTable, a cloud‐based platform for creating and sharing relational databases. Youth engagement was documented in each research stage (Figure 3). We recorded meeting minutes, project notes, PYA and YPAG suggestions, youth actions and capacity‐building insights. PYAs entered meeting notes and suggestions in AirTable, tagging relevant Steering Committee members to review and implement recommendations (summaries were shared with YPAGs and the broader Steering Committee as necessary). Additionally, PYAs and their research teams at each institution were interviewed to discuss their experiences, capacity‐building recommendations and youth engagement in decisions. These interviews were transcribed, summarized and shared with the Steering Committee.

#### 2.4.2 Data Analysis

##### 2.4.2.1 Categorizing Engagement

We applied Hart's ladder to delineate the level of youth engagement in our study. We adapted Hart's model by categorizing levels of youth participation into four categories—substantial, moderate, minimal and no engagement—based on the youths’ decision‐making power in the research process (see Figure 1). Substantial engagement was characterized by youth‐initiated and ‐directed activities, with adults fostering an enabling environment, allowing youth significant decision‐making power and leadership opportunities. Moderate engagement involved adult‐initiated activities where decision‐making was shared between youths and adults. Minimal engagement occurred when youths were consulted for their opinions, assigned specific roles or informed about research activities, but without any decision‐making authority. No engagement was defined by a complete lack of participatory methods or activities in the research process.

##### 2.4.2.2 Directed Content Analysis Process Using Hart's Ladder

Although Hart's ladder was intended to be applied as a flexible tool, it has not always been used as such, and our application seeks to illustrate the fluidity of youth engagement. Higher rungs are associated with greater youth involvement in project decisions, consistent engagement or solicitation of feedback and incorporation of their feedback. Conversely, lower rungs on the ladder correspond to reduced youth involvement in decisions‐making and less incorporation of youth feedback in project decisions.

Using a directed content analysis [40], we systematically described and evaluated our youth–adult participatory approach by analysing data (i.e., text) collected through AirTable, meeting minutes, project notes and transcripts of interviews conducted with PYAs. The research team used Airtable to collect, manage and organize data from PYAs, YPAGs, IYP and GYP. Airtable enabled collaborative data organization and time‐stamped entries for tracking feedback and project decision time points. Drawing from Hart's ladder, we predefined a coding scheme based on the ladder rungs that corresponded to different research stages. In this structured analysis, data within each research stage were examined. We identified text from meeting minutes, project notes and interviews where project decisions were recorded to examine when and how youth feedback was solicited and the extent to which this feedback influenced project decision‐making. We present these results through detailed descriptions of each research stage. Youth advisors in the MindKind study were given the opportunity to offer feedback on findings.

#### 2.4.3 Ethical Statement

This study protocol was approved by ethics committees at each of the study locations, namely, the United States (WIRB #20212067), the United Kingdom (University of Cambridge, Department of Psychology Research Ethics Committee: Ref. PRE.2021.031 and University of Oxford: Ref R73366/RE00), South Africa (Walter Sisulu University #029/2021 and the Department of Higher Education and Training) and India (India Law Society #ILS/242/2021 and Health Ministry Screening Committee). PYAs and youth panel members did not provide consent for their membership in youth advisory boards. Instead, they were part of the study staff and were compensated for their time and participation. This was to ensure that they could participate as equal decision‐makers in guiding the research process.

## 3 Results

### 3.1 PYAs

The three PYAs, all women, came from diverse professional backgrounds, including finance, sexual and reproductive health, and psychology. They had different levels of research experience. The first PYA who was hired had extensive research experience and was involved in formulation of MindKind's overarching goals and approach. The second PYA, who was hired at a university, had no prior research experience. The third PYA worked closely with her site supervisor and had prior youth facilitation experience but lacked formal research training. They all underwent an onboarding process combining human resources training, group and one‐to‐one discussions with their teams and individual researchers and on‐the‐job training with regular feedback.

### 3.2 Youth Panels

PYAs established and onboarded in‐country panels at different times: India in December 2020, the United Kingdom in March 2021 and South Africa in April 2021. Recruitment methods and panel composition varied by site due to differences in the legal age of consent. The United Kingdom panel had over 250 applicants aged 16–24 years from diverse backgrounds, whereas Indian and South African panels included youth aged 18–24 years primarily engaged in education or employment. Engagement strategies also varied by site, with different meeting frequencies and asynchronous engagement methods like WhatsApp groups. YPAG members underwent onboarding led by PYAs, including two to three orientation sessions on MindKind's objectives and design, with the option to explore mental health research topics as well capacity‐building opportunities.

### 3.3 Youth Engagement

Throughout the study, youth engagement ranged from Rung 4 (‘Assign but informed’) to Rung 7 (‘Youth initiated and directed’) of Hart's ladder [16] (Table 1). Initially, youth panels focused on formative research in data governance, application strategies and selecting ‘active ingredients’ [41] for databank measurement. At Rung 4, youth provided informed contributions but did not initiate projects themselves [16]. All YPAGs started at this level. With the PYAs serving as voting members of the Steering Committee, their involvement mainly spanned Rungs 5 to 7, where decision‐making was collaborative between adults and youth, or youth‐led.

**Table 1:** Youth engagement across MindKind study research stages.

MindKind study research stages	Rung on Hart's ladder	Youth engagement category
*Cross‐cutting research process*: *Youth engagement in overall study communication*	Rungs 5: Youth actively participated in designing communication strategies to improve the project structure, but also faced issues like lack of transparency and diminished trust.	Moderate engagement
*Stage 0—PYA onboarding*	Rung 4: PYAs were integrated into primarily adult‐led activities, with limited input on the onboarding process.	Minimal engagement
*Stage 1—Formative research: Data governance*	Rung 5: Youth input on data governance models enhanced the study's feasibility phase without fundamentally changing the design.	Moderate engagement
*Stage 1—Formative research: Application engagement strategies*	Rung 4: Youth suggestions mostly unincorporated into immediate decisions; considered only post‐decision.	Minimal engagement
*Stage 2—Quantitative study: Participant recruitment*	Rung 5: Youth actively participated in designing recruitment strategies.	Moderate engagement
*Stage 3—Qualitative study design and implementation*	Rung 6: Youth significantly influenced the structure of deliberative democracy sessions, as well as the educational materials and videos.	Moderate engagement
*Stage 3—Qualitative study design and implementation*	Rung 7: PYAs facilitated and led in‐country and multinational focus group discussions on deliberative democracy topics.	Substantial engagement

During the first 6 months, youth involvement in the MindKind project was minimal, with adults leading most decisions and youth roles still being established. This phase was necessary to define project goals and structure, but limited youth participation. Initially, some youth felt that their mental health experiences were underutilized due to sparse interactions with researchers. Although plans to integrate some youth suggestions for the MindKind app were postponed, moderate engagement occurred later in formative research stages. Additionally, there were few opportunities for the substantial engagement at Rungs 6 and 7 [16] demonstrated by youth feedback during the qualitative study shaping governance models and materials, and the creation of a video with YPAGs to improve the databank's clarity and effectiveness.

Several challenges influenced the project's ability to engage youth at higher ladder rungs, including tight timelines, budget constraints and scope and feasibility issues. External actors (e.g., funding bodies) and time constraints guided significant decisions. The project was relatively short (about 20 months), with an ambitious scope, limiting design options. The project was eventually extended, with the agreement of the funder, due to the challenging timelines. For example, to streamline the study, it excluded youth without smartphones and focused on those with Android devices, given their global predominance. Specific youth suggestions for personalized app features were postponed due to resource and time constraints.

To illustrate the youth engagement throughout the MindKind study, we present different research stages that highlight opportunities and challenges. Communication throughout the study was an important aspect that intersected across research stages; therefore, it is presented as a cross‐cutting process. Our results highlight the following research stages: Cross‐cutting research process, Stage 0—PYA onboarding, Stage 1—Formative research, Stage 2—Quantitative study and Stage 3—Qualitative study design and implementation.

### 3.4 Cross‐Cutting Research Process: Communication Strategies to Promote Youth Engagement: Moderate Engagement

Communication between youth and project team members varied across the project, raising issues like lack of transparency and diminished trust. PYAs raised concerns with their country project teams or the Steering Committee. Most sites held regular team meetings weekly, both in person and virtually, supplemented by informal check‐ins. PYAs frequently discussed communication challenges with supervisors, and one PYA initiated informal ‘crisis meetings’ on Zoom for planning and guidance. Another held quarterly mutual feedback sessions with their supervisor. For escalated issues, such as reimbursement, PYAs consulted the site principal investigator.

A significant outcome of PYA feedback was the creation of a community norms guide and safeguarding protocol to protect all participants from potential harms. This policy, developed with PYA input, was anonymously reviewed, approved by the Steering Committee and endorsed by MindKind leadership. Mid‐project, this policy and an anonymous feedback tool, monitored by designated leaders, were implemented, demonstrating PYA influence on project policy and structure. In response to feedback about transparency and engagement in Steering Committee meetings, the study coordinator began sending weekly digest emails that included discussion topics, meeting links, updates and a ‘You are Here’ section to indicate the project's progress, enhancing project transparency and engagement. Additionally, site‐specific meetings discussed post‐project career goals and occasionally featured PYAs as guest speakers at departmental meetings. Despite communication challenges, youth remained engaged, and some suggestions were successfully incorporated into the project structure, aligning with Rung 5 on Hart's Ladder.

### 3.5 Stage 0—PYA Onboarding: Minimal Engagement

Onboarding experiences varied across three sites—from structured, supportive environments to more independent, challenging ones. At two sites, PYAs were recruited early, benefiting from formal onboarding processes that included regular meetings with supervisors, relevant readings and collaboration on other projects. This comprehensive onboarding improved their familiarity with MindKind and broader research practices, equipping them with skills like facilitation and grounding them in decision‐making roles. However, the third site had a less structured onboarding approach, resulting in isolation and delayed engagement for the PYA due to late recruitment and pandemic constraints.

In the early stages, PYAs were integrated into primarily adult‐led activities with limited input on the onboarding process, aligning this stage with Rung 4 on Hart's Ladder. All the PYAs reported initially feeling overwhelmed from high expectations and inexperience in managing YPAG meetings on topics like data governance. PYAs gradually overcame these challenges through collaboration with project teams, which enhanced their capabilities and led to greater autonomy and ownership of tasks.

### 3.6 Stage 1—Formative Research: Youth Engagement in Data Governance and Application Strategies

Youth engagement in the formative research stage involved researchers consulting with youth panel members about the project scope and focus, particularly in areas like data governance models and application engagement strategies. During the protocol development phase, the PYAs, GYP and the India YPAG were consulted to review the proposed methodology and approach. Notably, the South Africa and UK YPAGs were not established at this time.

#### 3.6.1 Data Governance—Moderate Engagement

Youth panel members from the GYP and India's YPAG were moderately engaged in data governance discussions for the MindKind study's quantitative research arm. Their insights helped shape the quantitative study design, with none of their suggestions being rejected. Most feedback was integrated, whereas some was deferred due to scope limitations or logistical constraints (refer to Table 2).

**Table 2:** Incorporation of youth recommendations into the MindKind study.

MindKind study research stages	Example youth recommendation(s)	Incorporated (Y/N)
*Cross‐cutting research process: Youth engagement in overall study communication*	− Safeguarding policy (PYA) − Weekly project emails (PYA) − Anonymous feedback platform (PYA)	Y
*Stage 0—PYA onboarding*	N/A	N/A
*Stage 1—Formative research: Data governance*	− Data should be managed by a trusted, knowledgeable community manager (GYP, YPAG) − Suggestion to not restrict data access to the Global North (GYP)	Y
*Stage 1—Formative research: Application engagement strategies*	− Inclusion of self‐reflection prompts, push notifications and changing prompt tone in app design (YPAG)	N
*Stage 2—Quantitative study: Participant recruitment*	− Use of existing social media platforms that young people already use to build communities or connections like Slack (YPAG)	N
*Stage 2—Quantitative study: Participant recruitment*	− QR codes on physical posters in schools or youth clubs (YPAG) − Advisors/team to go into local schools and run sign‐up sessions with young people (YPAG)	Y
*Stage 3—Qualitative study design and implementation*	− Feedback on deliberative democracy videos (YPAG)	Y

Discussions primarily revolved around data access and permissible research activities. Youth panels often reached consensus on governance models, which were then presented to participants in the quantitative study. In India, YPAG members unanimously advocated for universal data access to improve mental health awareness and services. Similarly, the GYP advised against restricting access to the Global North. All youth emphasized ethical considerations, endorsing training in ethical conduct and the implementation of safeguarding measures. Both groups recommended appointing a trusted and knowledgeable community manager for data handling, insisting that institutions should cover data management costs. They also supported anonymizing data through a reconstructed data set to safeguard against the loss of original data, enhancing participant engagement in this formative phase of the research.

The Steering Committee integrated this feedback into the quantitative study protocol. This input was shared with the technology team, aiding in progressing the study beyond its feasibility stage. This level of engagement corresponds to Rung 5 on Hart's Ladder, reflecting moderate engagement. Youth input on data governance models was incorporated into the study design, as it did not significantly alter the study but enriched the feasibility phase.

#### 3.6.2 Application Engagement Strategies*—* Minimal Engagement

In the project's first 6 months, youth panel members had minimal involvement in suggesting features to improve engagement with the MindKind app. They proposed suggestions such as chatroom, positive affirmations, a habit tracker, a community forum, thank‐you messages for survey participation, reminder messages and feedback on app usage insights. Youth emphasized the importance of app personalization, arguing that ‘mental health is not one size fits all’.

However, many of these suggestions, including self‐reflection prompts, push notifications and varied prompt tones, were not adopted due to constraints related to the project's scope, budget and timeline. For instance, the project team noted the challenges, stating, ‘As for personalization… this will require engineering we currently do not have support for but will mark as a future feature’. Proposals to use social media for community building and engaging local professionals were rejected due to privacy concerns.

Limited incorporation of youth feedback places this stage at minimal engagement at Rung 4 on Hart's ladder. Although the youth panel was informed about the app's purpose and consulted for their opinions on engagement strategies, most of their input was either overlooked or solicited after key decisions had been made, leading them to perceive that their voices were underrepresented. Despite this, their suggestions were relayed to the funder for potential inclusion in future project phases. Reflecting on the process, YPAG members voiced concerns that their recommendations, particularly those enhancing app engagement, were not adequately considered.

### 3.7 Stage 2*—*Quantitative Study: Youth Engagement in Participant Recruitment*—*Moderate Engagement

Youth actively participated in recruiting participants across their respective countries, helping to meet the study goal of recruiting 1500 young people per site for the quantitative study arm. Each site had its own tailored recruitment strategy, using social networks and online platforms, though implementation varied by location.

Before recruitment began, youth were tasked with designing promotional materials. During YPAG meetings, PYAs presented draft materials, gathering input on graphic design, branding and poster creation. In the United Kingdom, youth suggestions such as incorporating QR codes into posters in schools and organizing local sign‐up sessions were implemented. The India YPAG recommended ‘…increasing font size, making the language more youth friendly, restructuring logos/images to help highlight text…’. Additionally, six YPAG members volunteered for a video series on social media, influencing the content and format of recruitment videos.

PYAs facilitated YPAG recruitment discussions, gathered feedback and guided decision‐making within the Steering Committee, implementing innovative strategies contributed by the youth, as detailed in Table 2. Data on recruitment from the South African panel are limited as they were primarily recruited from university campuses and faced challenges like ‘load shedding’ (i.e., planned electricity outages), which affected engagement.

Other suggestions from the GYP, such as using social platforms like Slack and Discord for community building, were deferred due to time and resource constraints. This moderate level of youth engagement corresponds to Rung 5 on Hart's Ladder, highlighting some youth influence on the recruitment of study participants.

### 3.8 Stage 3*—*Qualitative Study Design and Implementation: Youth Engagement in Designing Deliberative Democracy Sessions*—*Moderate Engagement

During the qualitative study design phase, youth panel members significantly contributed to developing study methodologies, which included deliberative democracy sessions in each country (refer to Sieberts et al. [37] for details) [36]. They influenced the creation of educational materials and governance models.

MindKind team members responsible for qualitative data collection also held capacity‐building sessions to educate youth panel members on qualitative research methods. These sessions covered the basics of qualitative research, data collection and analysis. Through interactive exercises, participants practiced interpreting transcripts, performing directed content analysis, identifying key themes and drawing conclusions. Examples of qualitative studies were also provided to enhance their understanding of the methods. Youth participants reported feeling highly engaged and better prepared for conducting qualitative research.

Before starting the qualitative study, the project team developed educational materials and recorded two video modules for participants to review before deliberative sessions. YPAGs and PYAs contributed to discussions on governance model examples and assisted in creating informational videos and educational content.

India's YPAG members enriched the data governance materials by suggesting the inclusion of health data‐related stories like the Aarogya Setu app and Aadhar card (the Aarogya Setu is a mobile application developed by the Indian government to spread awareness of COVID‐19 and to connect the people of India to essential health services. The Aadhar card is a 12‐digit unique biometric identification system available to Indian citizens and resident foreign nationals), emphasizing concerns about data security and privacy. Many mentioned WhatsApp's updated privacy policy, which prompted a shift among young users towards alternative messaging apps like Telegram and Signal. During a discussion on the deliberative democracy video, a UK YPAG member noted, ‘I think social media apps have the biggest conversation around data governance right now’. This perspective was integrated into the informational videos and added to the group discussion topics. In the qualitative study design, youth engaged at Rung 6, collaboratively fulfilling their roles. Youth contributions directly shaped the deliberative sessions, educational materials and videos.

#### 3.8.1 Deliberative Democracy Sessions Implementation—Substantial Engagement

Young people were instrumental in facilitating the deliberative democracy sessions, which were conducted remotely via Zoom due to the COVID‐19 pandemic. PYAs primarily led these sessions using a guide and conducting ‘mock sessions’ with YPAGs to prepare for data collection. Besides being trained as facilitators, PYAs engaged YPAGs in planning these sessions. In the qualitative study implementation, the PYAs engaged at Rung 7, facilitating and leading deliberative democracy sessions.

## 4 Discussion

Our study assessed youth engagement in the MindKind project, which aimed to establish a global mental health databank using digital data from young people in India, South Africa and the United Kingdom. Engagement levels varied, sometimes reaching higher rungs on Hart's ladder where youths shared decision‐making roles, but occasionally fell to minimal levels. Although contributions from youth on data governance models, quantitative study recruitment and qualitative study design were largely integrated, engagement declined notably as the project transitioned from design and data collection to analysis and dissemination. This is notable, as no project data on youth engagement during the analysis phase were recorded in AirTable. This trend underscores a broader issue in maintaining consistent youth engagement across all research phases, indicating a need for strategies that ensure sustained engagement, particularly in the later stages of research. This aligns with findings from studies on youth participation in violence‐related research, which also highlight significant gaps in youth engagement in analysis and dissemination [42].

Applying Hart's Ladder revealed not only varying levels of engagement but also shifting power dynamics in youth‐involved research. In the MindKind study, youth engagement fluctuated between minimal and substantial levels due to underlying power structures—how decisions were made, who controlled the resources and whose expertise were prioritized. Youth engagement peaked when their input was deemed feasible within the project's scope and budget, but diminished when project structure or financial support limited their involvement.

Youth engagement often reflected their direct lived experiences, leading to greater input in data governance and recruitment strategies where their personal experiences were applicable. Conversely, their engagement in technical stages like data analysis was limited due to their lack of knowledge, prioritizing researcher expertise instead. Notably, youth panels were not reconvened after data collection to discuss findings, representing a lost opportunity to enrich the research with their diverse perspectives, especially in interpreting results. Although some studies show that youth participation in data analysis can deepen the interpretation of findings, ensuring alignment with their views [15], concerns remain about potential biases, such as leading questions or personal biases influencing the analysis [43]. In our study, the absence of youth in the analysis and dissemination phases can be attributed to the project's structure, which, with its predefined phases and budgets, did not allow for extensive youth engagement past the data collection stages.

The funding structure and project timelines significantly influenced youth engagement levels. Most of the funding, excluding personnel costs, was allocated to the ethics clearance and primary data collection phases. This often led to reduced youth engagement in later research stages, such as data analysis, interpretation and dissemination. The need to adhere to tight timelines and budget constraints often prioritized efficiency over sustained youth engagement, reinforcing adult‐centric power dynamics typical of traditional research paradigms.

Moreover, the fluctuating levels of youth engagement in the MindKind study suggest the need for a more flexible ‘rope ladder’ framework [44]. This approach adapts to the dynamic needs of youth, offering a more inclusive and personalized method of engagement with multiple entry points and pathways. It reflects the evolving experiences and perspectives of youth in the research process, providing greater flexibility and adaptability for involvement. Continuous reflection on achievements and challenges during the MindKind project, along with the Steering Committee's commitment to youth engagement, encouraged young people to assume more substantial co‐design responsibilities and shared decision‐making.

The decision‐making structure in our study was notably unique. PYAs played an active role on the Steering Committee, influencing key project decisions. Throughout the project, adaptations such as developing safeguarding policies and implementing regular project emails were made to meet youth needs. Youth were involved in various research stages, including data collection and knowledge exchange, aligning with the flexibility, mentorship, authentic decision‐making and reciprocal learning principles noted in the McCabe et al. [15] review. YPAG members engaged in workshops, consultations and focus groups, consistent with co‐design practices for digital mental health technologies outlined in the Jones et al. [5] review.

Youth engagement was complicated by the COVID‐19 pandemic, and yet, it also provided opportunities for virtual collaboration, enabling shared decision‐making and co‐design [45]. Although impact evaluations [46] and more routine evaluation of co‐design are necessary [38], empowering young voices not only challenges the status quo but also enables youth with lived experiences to play active roles in relevant research decision‐making. Recent studies refer to this as experience‐based co‐design [47], an approach to health and social system change that integrates participatory action research, narrative and learning theory and design thinking. In this context, Mulvale et al. [47] emphasized the importance of trust‐building, perspective‐sharing and developing a collective vision. Meaningful youth engagement in mental health research showcases the benefits of participatory methods, enhancing the design of interventions that align with youth realities. Active engagement also sharpens youth critical skills, such as thinking, communication, problem‐solving and collaboration, which are transferable to various life aspects, as seen in the MindKind study outcomes [38].

### 4.1 Challenges of Participatory Youth Engagement

The MindKind study needed an effective organizational structure and communication process to meet youth's informational and capacity‐building needs. We addressed this challenge by using a database (AirTable) and multiple feedback channels. However, learning from these challenges helped formulate proactive strategies for addressing future challenges, like workshops for manuscript writing and dissemination activities with youth.

Nevertheless, infrastructural challenges impacted youth engagement significantly, with load shedding, that is, planned electricity outages, and limited internet access among South African youth advisors hindering their involvement. Our challenges highlight the need for improved research infrastructure to support participatory research with youth, especially in lower‐resourced settings [38]. Our project's challenges are consistent with the broader co‐production literature, highlighting the need for increased flexibility, time, resources and equitable partnerships based on mutual respect, trust and reciprocity to achieve successful co‐production [48].

### 4.2 Implications and Recommendations

Engaging youth in mental health research is a transformative endeavour with the potential to challenge traditional power dynamics and amplify the voices of youth with lived experience of mental health challenges. The complexities introduced by the COVID‐19 pandemic also presented opportunities for collaboration on virtual platforms for shared decision‐making [38]. The MindKind study can serve as a blueprint for youth engagement, considering its project structure, challenges and adaptations. This study demonstrates the value of explicitly adopting a youth–adult participatory approach that establishes structures to promote youth exercise of their participatory rights. This fundamental principle underpins much of the youth engagement in the MindKind study, including acknowledging and addressing where such rights could not be exercised by youth.

### 4.3 Youth Engagement Recommendations

- *Rights‐based approach*: Integrate youth rights into the study, ensuring youth representation and voting rights on key decision‐making bodies like the Steering Committee.
- *Clear expectations and feedback tracking*: Establish and communicate clear expectations from the outset. Implement a systematic process for collecting feedback and reporting, helping youth understand their impact and fostering a sense of ownership. Regularly update youth on how their contributions are influencing the project, clearly communicating which insights are being incorporated and which are not. Use direct feedback mechanisms, such as personal communications or tools like AirTable.
- *Administrative and financial support*: Ensure timely and fair compensation for youth involvement, accommodating geographic and infrastructural variations. Such administrative and financial support must also extend to the project as a whole, to address key gaps in youth engagement practice, that is, funding to enable youth engagement in data analysis, interpretation of findings and dissemination of findings.

- *Recognize dynamic engagement and adopt responsive strategies*: Recognize and adapt to the evolving nature of youth engagement, due to factors like individual interests, cultural backgrounds, availability and personal development stages.
- *Remain flexible*: Changes in engagement levels and strategies may occur due to evolving project needs; maintain open communication with youth to continuously refine approaches. Consistently document outcomes to track the effectiveness of strategies and facilitate continuous improvement.
- *Implement, when possible, training opportunities and capacity building*: Offer training sessions that align with youth interests and needs to enhance skills, such as facilitation, collaboration, reflexivity and positionality training; also encourage youth to suggest additional topics.

### 4.4 Limitations

Our study has limitations, particularly in engaging youth at higher levels of Hart's Ladder. First, the fully remote interaction with youth panels limited our ability to sustain YPAG engagement, with differing time zones complicating global coordination. Second, delays in researcher feedback to youth panels hindered timely engagement, impacting youth motivation and participation. Third, minimal data were received from youth advisors and PYAs in South Africa due to significant regional infrastructural challenges, affecting our analysis of youth engagement in that context. Fourth, initially unclear expectations for youth roles during the first 6 months may have skewed perceptions of youth engagement. Lastly, although PYAs documented notes, feedback and suggestions from YPAG sessions, the lack of direct quotes and limited data points from a small number of youth advisors constrained the depth of perspectives shared. The minimal direct quotes were also a result of efforts to maintain confidentiality. Despite these limitations, to our knowledge, this is the first analysis in global mental health research to explore the shifting nature of youth engagement and the allocation of power across research stages.

### 4.5 Conclusion

Our study sheds light on youth–adult participatory research with young people in the MindKind study, aiming to establish a global mental health databank. Youth engagement in project decision‐making evolved over time, with youth moving up and down rungs on Hart's Ladder, demonstrating their co‐design responsibilities. Their involvement influenced key study outcomes, particularly in quantitative recruitment activities, data governance models and qualitative study design and implementation. However, youth engagement declined in data analysis, interpretation of findings and dissemination stages. Despite challenges stemming from project timelines and funding constraints, the study showcased the potential of youth–adult partnership models. Youth engagement holds significant promise, not only amplifying marginalized voices but also fostering skill development, leadership qualities and promoting inclusivity. Effective youth engagement demands dynamic strategies, transparent communication and mutual respect to shape research outcomes that authentically address diverse perspectives and mental health experiences.

## Author Contributions

Conceptualization: Augustina Mensa‐Kwao and Jennifer Velloza. Methodology: Augustina Mensa‐Kwao, Lakshmi Neelakantan, and Pamela Y. Collins. Data curation: Emily Bampton, Swetha Ranganathan, Refiloe Sibisi, and The MindKind Consortium. Software: The MindKind Consortium. Investigation: Augustina Mensa‐Kwao, Lakshmi Neelakantan, Emily Bampton, Swetha Ranganathan, Refiloe Sibisi, and The MindKind Consortium. Validation: Augustina Mensa‐Kwao, Lakshmi Neelakantan, and Pamela Y. Collins. Formal analysis: Augustina Mensa‐Kwao, and Lakshmi Neelakantan. Supervision: Pamela Y. Collins. Funding acquisition: Megan Doerr, Tamsin Jane Ford, The MindKind Consortium (Mina Fazel, Gillian Finchilescu, Melvyn Freeman, Zukiswa Zingela, Soumitra Pathare), and Pamela Y. Collins. Visualization: Augustina Mensa‐Kwao. Project administration: Megan Doerr, Tamsin Jane Ford, Christine Suver, The MindKind Consortium, and Pamela Y. Collins. Writing–original draft: Augustina Mensa‐Kwao, Lakshmi Neelakantan, and Pamela Y. Collins. Writing–review & editing: Augustina Mensa‐Kwao, Lakshmi Neelakantan, Jennifer Velloza, Emily Bampton, Swetha Ranganathan, Refiloe Sibisi, Joshua Bowes, Lilliana Buonasorte, Damian Omari Juma, Manasa Veluvali, Megan Doerr, Tamsin Jane Ford, Christine Suver, Carly Marten, The MindKind Consortium, and Pamela Y. Collins.

## Conflicts of Interest

The authors declare no conflicts of interest.

## Data Availability

Data used in this manuscript are not publicly available due to their confidential nature.
